# Setting research priorities for maternal, newborn, child health and nutrition in India by engaging experts from 256 indigenous institutions contributing over 4000 research ideas: a CHNRI exercise by ICMR and INCLEN

**DOI:** 10.7189/jogh.07.011003

**Published:** 2017-06

**Authors:** Narendra K Arora, Archisman Mohapatra, Hema S Gopalan, Kerri Wazny, Vasantha Thavaraj, Reeta Rasaily, Manoj K Das, Meenu Maheshwari, Rajiv Bahl, Shamim A Qazi, Robert E Black, Igor Rudan

**Affiliations:** 1 The INCLEN Trust International, New Delhi, India; 2 Centre for Global Health Research, Usher Institute for Population Health Sciences and Informatics, University of Edinburgh, Scotland, UK; 3 The Indian Council of Medical Research, New Delhi, India; 4 World Health Organization, Geneva, Switzerland; 5 Johns Hopkins Bloomberg School of Public Health, Baltimore, Maryland, USA

## Abstract

**Background:**

Health research in low– and middle– income countries (LMICs) is often driven by donor priorities rather than by the needs of the countries where the research takes place. This lack of alignment of donor’s priorities with local research need may be one of the reasons why countries fail to achieve set goals for population health and nutrition. India has a high burden of morbidity and mortality in women, children and infants. In order to look forward toward the Sustainable Development Goals, the Indian Council of Medical Research (ICMR) and the INCLEN Trust International (INCLEN) employed the Child Health and Nutrition Research Initiative’s (CHNRI) research priority setting method for maternal, neonatal, child health and nutrition with the timeline of 2016–2025. The exercise was the largest to–date use of the CHNRI methodology, both in terms of participants and ideas generated and also expanded on the methodology.

**Methods:**

CHNRI is a crowdsourcing–based exercise that involves using the collective intelligence of a group of stakeholders, usually researchers, to generate and score research options against a set of criteria. This paper reports on a large umbrella CHNRI that was divided into four theme–specific CHNRIs (maternal, newborn, child health and nutrition). A National Steering Group oversaw the exercise and four theme–specific Research Sub–Committees technically supported finalizing the scoring criteria and refinement of research ideas for the respective thematic areas. The exercise engaged participants from 256 institutions across India – 4003 research ideas were generated from 498 experts which were consolidated into 373 research options (maternal health: 122; newborn health: 56; child health: 101; nutrition: 94); 893 experts scored these against five criteria (answerability, relevance, equity, innovation and out–of–box thinking, investment on research). Relative weights to the criteria were assigned by 79 members from the Larger Reference Group. Given India’s diversity, priorities were identified at national and three regional levels: (i) the Empowered Action Group (EAG) and North–Eastern States; (ii) States and Union territories in Northern India (including West Bengal); and (iii) States and Union territories in Southern and Western parts of India.

**Conclusions:**

The exercise leveraged the inherent flexibility of the CHNRI method in multiple ways. It expanded on the CHNRI methodology enabling analyses for identification of research priorities at national and regional levels. However, prioritization of research options are only valuable if they are put to use, and we hope that donors will take advantage of this prioritized list of research options.

*“Today’s health research is tomorrow’s health service”* [1]. If the research agenda is not aligned to local needs and context, it can perpetuate disharmony, inequity and inefficiency in health services and contribute to lack of attainment of policy goals [2,3]. Given that the scope of research in health and nutrition is ever–expanding and far exceeds the available resources, relative prioritization among competing research options is imperative. This is difficult, liable to subjectivity and vulnerable to being funder–driven [4]. Prioritization using a systematic, transparent, objective and inclusive process could help policy makers and research funding agencies in making their investment decisions more co–aligned, efficient and impactful [2].

The 10–90 report of the Commission on Health Research for Development (1990) emphasized on the prevailing mismatch between local health research needs and the quantum and patterns of fund allocation, particularly in low– and middle– income countries (LMICs) [5]. Between 1990 and 2005, following the 10–90 report, several attempts were made at developing structured and objective methods to identify priorities. Prominent among these, were: (i) the Ad Hoc Committee on Health Research Relating to Future Intervention Options, 1996; (ii) The Council on Health Research and Development (COHRED); (iii) the Essential National Health Research and Priority Setting (ENHR), 1996–2000; (iv) The Grand Challenges in Global Health in 2003; and, (v) the Combined Approach Matrix (CAM) tool by the Global Forum for Health Research, 1999–2004 [3,6]. In 2006–07, the Child Health and Nutrition Research Initiative (CHNRI), informed by weaknesses in existing processes, developed a flexible yet systematic method for setting research priorities, called the CHNRI method. The CHNRI method has become increasingly popular and to date, over 50 CHNRI research priority setting exercises have been reported [7]. This method recognizes research priority setting as a multi–dimensional and multi–stakeholder decision–making process. It balances immediate contextual translational needs (the ‘delivery’ and ‘development’ instruments of research) with need for generation of new knowledge through long–term investment (“description” and “discovery”). The CHNRI method systematically delegates, ie, “crowdsources,” [8] the task of prioritization to the various constituencies of stakeholders (end–users of health research funding) [9]. Crowdsourcing is the use of collective wisdom or collective tasks for the benefit of an individual and or an organization, such as to solve a problem or complete a task [10]. The CHNRI method has been shown to be effective at the national level wherein input from local stakeholders can influence research investment policies [11].

India is the second most populous country in the world with many pressing health problems that, in fact, hugely determine the global health statistics. Maternal, neonatal, child health and nutrition (MNCHN) together contribute to the largest burden of disease in India. Public health research decisions in India have traditionally been guided by a small group of experts who are located mostly in the metropolis and are constrained by individual and organizational preferences. In 2011, in response to the seemingly unachievable Millennium Development Goals 4 and 5 (MDG4, MDG5), National Health Mission goals, and the upcoming Sustainable Development Goals 2030, the Indian Council for Medical Research (ICMR; the apex institution for medical research in India) and the INCLEN Trust International (INCLEN; which was the CHNRI Secretariat since 2010) came together to undertake this nationwide research priority setting exercise for MNCHN using the CHNRI methodology. Newborns, children (0–18 years), and reproductive age women (15–49 years, including pregnant women and lactating mothers) were identified to be the target population for prioritization along the life–course continuum. India has large population diversity along with regional– and state–level heterogeneity in governance, program performance, socio–cultural milieu and economics. Hence, it was decided that research priorities would be identified at national and sub–national (regional) levels with a 10–year reference time period (2016–2025) and through inclusion of a large number of stakeholders for representativeness.

## METHODS

The ICMR–INCLEN National Research Priority Setting (RPS) exercise was completed between 2012 and 2016. The exercise was coordinated by the RPS project management team at the Executive Office of INCLEN, New Delhi. The team had experts in the four core MNCHN disciplines (pediatrics, obstetrics and gynecology, community medicine, and public health nutrition) and was multilingual and hence, able to communicate and engage participants from across the country.

States and union territories were grouped into three regions in order to enable sub–national priorities. The three regions were: (i) Empowered Action Group (EAG) States (Rajasthan, Madhya Pradesh, Chattisgarh, Odisha, Jharkhand, Bihar, Uttar Pradesh and Uttarakhand) and North–Eastern (NE) States (Sikkim, Assam, Meghalaya, Tripura, Mizoram, Manipur, Nagaland, Arunachal Pradesh); (The Government of India has identified eight states with poor health and development indicators as EAG states for focused action. EAG and NE states share similarities in MNCHN contexts and program performance.); (ii) Northern states and Union territories (Jammu & Kashmir, Punjab, Himachal Pradesh, Haryana, Chandigarh, Delhi, and West Bengal); and (iii) States and Union Territories in Southern and Western part of the country (Kerala, Tamil Nadu, Karnataka, Andhra Pradesh and Telangana, Maharashtra, Gujarat, Goa, Puducherry).

Four key structures were created to accomplish the task, outlined as follows.

### 1. The National Steering Group (NSG)

The NSG was the highest body for policy making and oversight for the exercise. Its responsibilities included (i) setting the rationale and contour of the MNCHN research themes; (ii) establishment of research sub–committees (RSCs); (iii) critical review, interpretation and endorsement of the results of the exercise; and, (iv) dissemination of the final national and regional research priorities. The NSG was co–chaired by the Secretary, Department of Health Research (DHR) & Director General (DG–ICMR) and Executive Director of INCLEN. It included key officials from the Ministry of Health & Family Welfare (National Health Mission, Child Health, Maternal Health and Nutrition divisions, Directorate General of Health Services and DHR–ICMR), Ministry of Women and Child Development (Integrated Child Development Services, Food and Nutrition Board), and Ministry of Science and Technology (Department of Biotechnology, Department of Science & Technology). Its membership also included invited subject experts and representatives of national and international donors and multilateral agencies. The chairs of all four RSCs were also members of the NSG (**Table 1**). Two NSG meetings were organized – the first (on 18th April 2013), at the initiation of the exercise to ratify the context **(****Box 1****)** and protocol, and the second (on 4th February 2016), at the conclusion to review, refine and finalize the results.

**Table 1:** Profile of the National Steering Group

Expertise	18 Apr 2013	4 Feb 2016
Policy–Decision Makers and Program Managers (MNCHN), Government of India	22	24
Multilateral/ Bilateral Donor Agencies/Foundation – Funders	15	19
Technical Experts (MNCHN)	29	21
State Program Managers (ICDS, NRHM, Directorate of Health Services)	9	11
Biomedical Journal Editors	3	3
**Total**	**78**	**78**

Box 1 **Context of the INCLEN ICMR national research priority setting exercise in maternal, newborn, children health and nutrition**

**Purpose:** Priority setting in maternal, newborn, and child health and nutrition for efficient and rewarding investment in research using a systematic, transparent, inclusive, objective and quantitative method.

**Target population:** Women of reproductive age (15–49 years) including pregnant and lactating women, newborns (0–28 days), under–five children (0–59 months) and children up to the age of 18 years.

**Geography:** Priorities at National and three Regional levels: Empowered Action Group States and North–Eastern States, States and Union Territories in Northern India, and those in Southern and Western India.

**Major areas of concern for research:** Conditions that together contributed to 75% of the mortality and morbidity burden in Maternal, Newborn, Child Health and Nutrition in India during 2012–2013 as per the available evidence and expert opinion.

**Time frame:** For the next ten years ie, 2016–2025 (with due consideration to unachieved Millennium Development Goals 1, 4 and 5, and National Health Mission targets and the challenge of preparing the national agenda for achieving forthcoming Sustainable Development Goals 2030).

**Stakeholder constituencies (operating in civil, public and private sectors, health and non–health sectors):** Researchers, professionals, public health functionaries, policy makers, communities and their leadership, civil society, donor agencies and industries.

**Translation and implementation context:** Public and private health systems of India and their existing as well as future programs, national and international institutions & organizations funding research, research environment in academic & research institutions.

### 2. The thematic Research Sub–Committees (RSCs)

A RSC was constituted for each of the four themes. The RSCs’ membership included technical experts (subject experts, basic scientists and public health specialists), social scientists, program specialists (health, and woman and child development), and donor agency representatives. Technical experts were identified through a literature search for active research contribution to respective MNCHN domains (**Table 2**). The RSCs participated in the crowdsourcing processes along with the nationwide network. They also helped in the iterative refinement and consolidation of the research options (ROs) and in finalizing the scoring criteria and their definitions. Respective RSCs presented the study findings to the second meeting of the NSG for review.

**Table 2:** Profile of research sub–committees and nation–wide network (1^st^ round of crowd–sourcing)

Group	Expertise	Theme (with components)							
**Maternal health**			**Newborn health**	**Child health**	**Nutrition**		**Total**	
**Mortality**	**Morbidity**	**Stillbirths**			**Maternal**	**Childhood**		
									
**Research Sub–Committee (RSC)**	Basic scientists*		1		1			1	3
	Dietitians and nutritionists						8	9	17
	Experts from ICMR institutes			1					1
	Nursing & midwifery experts			1					1
	Obstetricians and gynecologists	7	4	4					15
	Pediatricians and neonatologists				13	13			26
	Policy makers (Government of India)*	1	1		1	2			5
	Scientists from research institutes (public health and allied sciences)*	2		2	2	2	2	1	11
	State program managers*	3	1		1	2	1	2	10
	Technical Experts from donor agencies*	3	2	1	7	7	1	2	23
	**Sub–total**	**16**	**9**	**9**	**25**	**26**	**12**	**15**	**112**
**Nation–wide network (beyond RSCs)**	Agriculturists						3	5	8
	Basic scientists*	2	2	1	1	1	1	3	11
	Community medicine experts	44	45	42	39	38	61	35	304
	Dietitians & nutritionists						33	33	66
	Experts from ICMR Institutes*	8	9	9	10	12	6	4	58
	Miscellaneous*						1		1
	Nursing & midwifery experts	3	3	3					9
	Obstetricians and gynecologists	68	74	82	1		17		242
	Pediatricians and neonatologists				111	122		47	280
	Policy Makers (Government of India)*							2	2
	Scientists from research institutes (public health and allied sciences)*	7	4	4	7	8	3	5	38
	State program managers*	4	4		4	4	3	3	22
	Technical Experts from donor agencies*				2	1	5	5	13
	**Sub–total**	**136**	**141**	**141**	**175**	**186**	**133**	**142**	**1054**
	**Grand total**	**152**	**150**	**150**	**200**	**212**	**145**	**157**	**1166**

*The experts in these categories were requested to identify their theme/ component of expertise.

### 3. The Nationwide Network for crowd sourcing

A network was established with experts identified from institutions and departments across the country. Faculty/researchers from departments that were directly or indirectly engaged in work pertaining to MNCHN (eg,, obstetrics & gynecology, pediatrics, neonatology, community medicine, biochemistry, physiology, pathology, microbiology, midwifery, public health nutrition and home sciences, social sciences, statistics and demography, and agriculture) were contacted through their respective institutional heads. The effort was to secure similar proportion of faculty members/researchers with more than 10 years of research or teaching experience (ie, ‘senior’ faculty) and those who are junior/middle level with 5–10 years of experience. National and zonal office–bearers of major professional associations in MNCHN (the Indian Academy of Pediatrics, the National Neonatology Forum, the Federation of Obstetrics and Gynecological Societies of India, the Indian Association of Preventive and Social Medicine, the Indian Public Health Association, the Nutrition Society of India, and the Indian Dietetic Association) were also contacted for participation. Central and state–level policy–makers and program managers were also invited to participate in the exercise. These were from departments of health and of women and child development. Experts were also identified through snow–balling and invitations in personal capacity.

The members in the nationwide network consented to be allocated into one of the four themes according to their expertise and publication history to achieve equitable regional and disciplinary representation in each theme. In this manner, for the first round of crowd sourcing, 1423 experts (including the 112 in the RSCs) were identified, of whom 1178 could be contacted. Of these, 12 declined to participate. Of the remaining 1166 experts (**Table 2**), 668 did not respond. Overall, 498 (42.3%) experts contributed research ideas. For the second round (scoring activity), 1536 experts were contacted (including those contacted during the first round) of which 15 declined, 628 did not respond/ logged in but did not score, and 893 (58.1%) participated. Overall, 256 institutions including medical colleges, ICMR institutions, research organizations, NGOs, state health departments and donor agencies participated in the two rounds for crowdsourcing (**Table 3**).

**Table 3:** Profile of participating institutions in the Nationwide Network

State/Union Territory	Medical colleges			ICMR institutions		Other public health research institutes	Non–governmental organizations	State departments (health and nutrition)	Donor agencies	TOTAL
Assam	3			1		1	2	1		8
Manipur	1									1
Meghalya	1							1		2
Nagaland								1		1
Odisha	7			1		3				11
Sikkim								1		1
Tripura	2							1		3
West Bengal	11			1		2				14
Chandigarh	2									2
Delhi	8			2		8	5		2	25
Haryana	1					1		1		3
Himachal Pradesh	2							1		3
Jammu & Kashmir	1							2		3
Punjab	5					3				8
Uttar Pradesh	14					1				15
Uttarakhand						1				1
Goa	1						1			2
Gujarat	10					2		1		13
Maharashtra	19			3		1	1	2		26
Rajasthan	14					1	2			17
Andhra Pradesh	15			1		6	1	2		25
Karnataka	15			1		1		1		18
Kerala	9					1		2		12
Puducherry	1			1						2
Tamil Nadu	8			3		3				14
Bihar	4							2	1	7
Chattisgarh	4									4
Jharkhand	1									1
Madhya Pradesh	10			1		3				14
**Grand total**		**169**	**15**		**38**		**12**	**19**	**3**	**256**

### 4. The Larger Reference Group (LRG)

Beyond 75% of CHNRI exercises published have not employed a LRG (majorly due to trouble composing the group). Of those that could, most have been conducted at a national level [7]. To incorporate broader societal perspectives and values within the exercise, we employed a LRG which was composed of policy decision makers (n = 24; Central and State politicians and bureaucrats from key Ministries, eg, Health and Family Welfare, Woman and Child Development, Human Resource Development), senior researchers (n = 17), MNCHN program managers from central and state governments (n = 24) and representatives from research funding organizations (n = 19). The LRG attributed relative weights to the scoring criteria which helped to generate criteria–weighed priority ranks for the ROs.

### Processes

**Figure 1:**
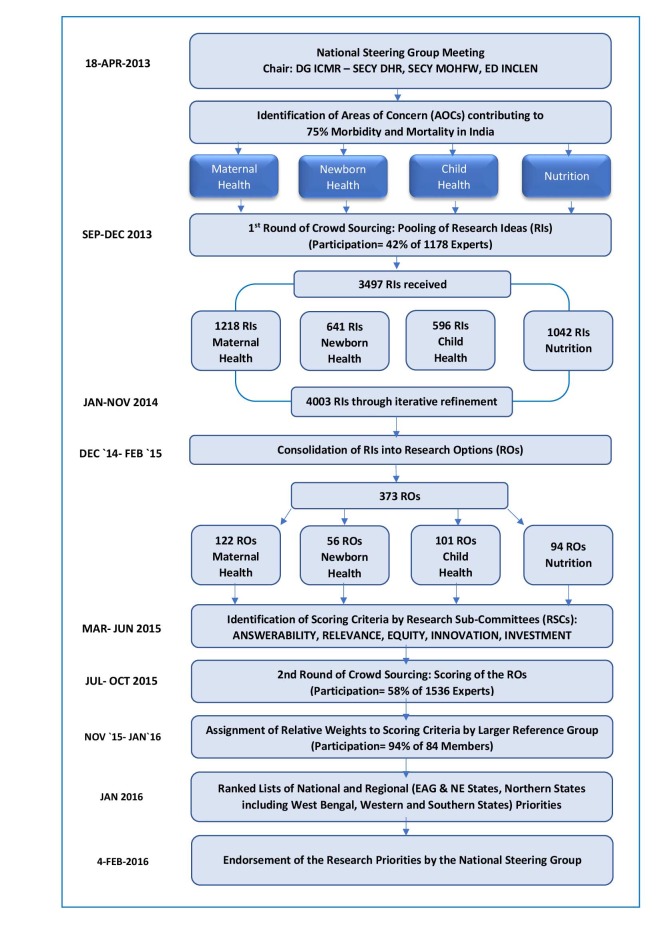
Sequence of activities undertaken in the ICMR–INCLEN National Research Priority Setting Exercise.

**Figure 1** shows the schematic flow of activities with timelines.

#### Review of literature and identification of areas of concern

Extensive review of literature on burden of MNCHN related conditions was done in 2012–13 with focus on Indian data. We searched published literature (indexed and non–indexed), Government of India’s policy documents and reports, program reviews and grey literature for the period of 1990 to 2012/2013. PubMed, CINAHL and Embase databases were searched. Based on the compendium of literature (available at: *www.inclentrust.org*), a draft list of “areas of concern” (AOCs) was prepared for each of the RPS themes and presented to the first meeting of the NSG for review. The AOCs accounted for conditions that collectively contributed to at least 75% of the mortality and morbidity burden in the respective theme.

The NSG suggested that maternal health encompassed three components viz., morbidity, mortality and stillbirths (most stillbirths occur in–utero and are thus are a maternal health concern). Similarly, it divided the nutrition theme into maternal and childhood nutrition components. The NSG advised to include three additional AOCs in each of the themes: “social determinants,” “impact and improvement of existing composite public health packages,” and “novel & innovative public health interventions.” The final approved list of AOCs under the four themes is presented in **Table 4**.

**Table 4:** Areas of concern finalized by the National Steering Group

Maternal Health Theme: Maternal Mortality Component	
**1**	Hemorrhage
**2**	Hypertensive disorders of pregnancy
**3**	Sepsis
**4**	Obstructed labor
**5**	Unsafe abortion
**6**	Anemia and other nutritional problems
**7**	Medical disorders in pregnancy [eg, chronic hypertension, epilepsy, liver disease, diabetes mellitus, renal disease, thyroid disease, lupus]
**8**	Malaria in pregnancy
**9**	Others (Please specify ________)
**10**	Social determinants of maternal mortality [eg, social isolation, stigmatization, marital disharmony, divorce, household dissolution, domestic violence, loss of community status; caste, religion, teenage pregnancy, cultural practices]
**11**	Economic [eg, impoverishment and poverty]
**12**	“Existing” composite public health packages with potential impact on MMR [eg, Janani Shishu Suraksha Karyakram (JSSK)]
**13**	“Novel & Innovative” composite public health packages with potential impact on MMR [eg, Innovative solutions to promote access to care]
**Maternal Health Theme: Maternal Morbidity Component**	
**1**	Severe acute maternal morbidities (SAMMs) and Near miss events
**2**	Post partum morbidities and long term disabilities [eg, obstetric fistula, utero–vaginal prolapse, urinary incontinence, dyspaerunia, infertility]
**3**	Post partum depression and psychosis
**4**	Strong fear of pregnancy and child birth
**5**	Social [eg, social isolation, stigmatization, marital disharmony, divorce, household dissolution, domestic violence, loss of community status; caste, religion, teenage pregnancy, cultural practices]
**6**	Economic [eg, impoverishment and poverty]
**7**	“Existing” composite public health packages with potential impact on maternal morbidity [eg, syndromic management of RTI & STI; Reproductive, maternal, newborn, child and adolescent health (RMNCH+A)]
**8**	“Novel & Innovative” composite public health packages with potential impact on maternal morbidity [eg, innovative solutions to promote access to care]
**Maternal Health Theme: Stillbirth Component**	
**1**	Maternal cause: Hypertensive disorders of pregnancy
**2**	Maternal cause: Maternal infections in pregnancy [eg, TORCH group of infections]
**3**	Maternal cause: Underlying chronic maternal illness [eg, chronic hypertension, epilepsy, liver disease, diabetes mellitus, renal disease, thyroid disease, lupus]
**4**	Maternal cause: Maternal malnutrition [eg, low maternal BMI, gestational diabetes]
**5**	Fetal cause: Intra uterine growth restriction
**6**	Fetal cause: Pre–term birth
**7**	Fetal cause: Congenital malformations
**8**	Intra partum cause: Acute hypoxic insult
**9**	Intra partum cause: Obstetric complications
**10**	Complications of placenta, cord and membranes
**11**	Unexplained [By known maternal, placental and fetal conditions]
**12**	Non-health factors [eg, Indoor air pollution, tobacco smoke]
**13**	Social determinants of stillbirths [eg, prevailing harmful traditional birth practices, lack of womens’ empowerment, poverty, illiteracy]
**14**	“Existing” composite public health packages with potential to influence stillbirths[eg, Janani Shishu Suraksha Karyakram (JSSK)]
**15**	“Novel & Innovative” composite public health packages with potential to influence stillbirths[eg, Innovative solutions to promote access to care]
**Child Health Theme:**	
**1**	Pneumonia (and ARI)
**2**	Diarrheal diseases
**3**	Measles and vaccine preventable diseases
**4**	Congenital anomalies
**5**	Malaria
**6**	Unintentional injuries
**7**	Acute bacterial sepsis
**8**	Meningitis/encephalitis
**9**	Other infections & parasitic diseases
**10**	Neuro–developmental disorders (NDD) [eg, early developmental delays, autism, speech & language disorders, intellectual disability, epilepsy, CP, neuro–motor impairment, audio–visual impairment]
**11**	Others (Please specify ____)
**12**	Social determinants of under 5 mortality rate [eg, immunization refusal, inappropriate feeding practices, poor health seeking behavior.]
**13**	“Existing” composite public health packages with potential impact on Under 5 Mortality Rate [eg, IMNCI, F-IMNCI, Reproductive, Maternal, Newborn, Child and Adolescent Health(RMNCH+A)]
**14**	“Novel & Innovative” composite public health packages with potential impact on Under–5 Mortality Rate [eg, Rashtriya Bal Swasthya Karyakram (RBSK) – Child Health Screening and Early Intervention Services]
**Newborn Health Theme:**	
**1**	Preterm birth
**2**	Neonatal sepsis including pneumonia
**3**	Birth asphyxia & Birth trauma
**4**	Congenital malformations
**5**	Others (Please specify ____)
**6**	Social determinants of NMR [eg, newborn care practices, poverty, poor health seeking behavior]
**7**	“Existing” composite public health packages with potential to influence neonatal morbidity and mortality [eg, IMNCI, Home based newborn care, Reproductive, maternal, newborn, child and adolescent health (RMNCH^+^A)]
**8**	“Novel & Innovative” composite public health packages with potential to influence neonatal morbidity and mortality [eg, Innovative solutions to promote access to care]
**Nutrition Theme: childhood nutrition component:**	
**1**	Protein energy malnutrition (PEM)
**2**	Low birth weight
**3**	Micro-nutrient deficiencies (iron/folic acid/zinc/iodine/Vitamin A)
**4**	Childhood overweight and obesity
**5**	Nutrition deficiency associated congenital malformations
**6**	Fetal and child nutrition and origin of adult chronic non–communicable diseases [eg, cardiovascular diseases, metabolic syndrome, obesity etc.]
**7**	Socio–cultural and economic determinants: time constraint with mothers entering into the work force
**8**	Socio–cultural and economic determinants: care and feeding practices
**9**	Socio–cultural and economic determinants: competing use of resources for goods and services other than nutrition/food
**10**	Socio–cultural and economic determinants: globalization & market forces influencing food habits
**11**	Socio–cultural and economic determinants: status of girl child and women in the community
**12**	Socio–cultural and economic determinants: use of pesticides & fertilizers
**13**	Socio–cultural and economic determinants: potable water, hygiene and sanitation
**14**	Socio–cultural and economic determinants: others (Please specify ____)
**15**	“Existing” composite public health packages with potential impact on Child nutrition [eg, ICDS, Mid-day Meal Program]
**16**	“Novel & Innovative” composite public health packages with potential impact on child nutrition [eg, Food fortification program, promotion of kitchen gardens/organic farming, deworming, convergent-innovation coalition to address issues of anemia, under-nutrition, obesity]
**Nutrition Theme: Maternal Nutrition Component**	
**1**	Anemia among women of reproductive age group
**2**	Iodine deficiency disorders among women
**3**	Vitamin D deficiency among women
**4**	Maternal overweight & obesity and other non-communicable diseases
**5**	Socio–cultural and economic determinants: time constraint with mothers entering into the work force
**6**	Socio–cultural and economic determinants: care and feeding practices
**7**	Socio–cultural and economic determinants: competing use of resources for goods and services other than nutrition/food
**8**	Socio–cultural and economic determinants: globalization & market forces influencing food habits
**9**	Socio–cultural and economic determinants: women’s status in the community, family structures and norms
**10**	Socio–cultural and economic determinants: others (Please specify ______)
**11**	“Existing” composite public health packages with potential impact on maternal nutrition
**12**	“Novel & Innovative” composite public health packages with potential impact on maternal nutrition

The NSG also suggested that all research ideas (RIs) be segregated into the four domains of research: (i) description (burden of disease, epidemiology, etiology and risk factors, biomarkers, pathophysiological descriptions); (ii) discovery (identification of novel pathways, discovery of novel clinical and public health interventions/package, technology inventions, discoveries and innovations); (iii) delivery (health policy and systems research, including program evaluation and implementation research); and (iv) development (improving the existing intervention, ie, design, deliverability, affordability and sustainability).

#### Crowdsourcing

##### First round of crowdsourcing

**Solicitation of research ideas (RIs) from the Nationwide Network:** An online software was designed by INCLEN for submission of RIs by the network. The software had seven separate electronic forms: maternal health (n = 3: mortality, morbidity & stillbirth); newborn health (n = 1); child health (n = 1); and nutrition (n = 2: maternal & child nutrition) themes]. The experts in the nationwide network and RSCs were sent an initial email and then contacted over the phone: (i) to sensitize them about the method of the research priority setting exercise; (ii) to provide them the context and scope of the exercise; and, (iii) the provide them with the purpose of the first round of crowdsourcing. Each participant was provided with an individualized log–in username and password for the dedicated software. The participant could log in to only one of the seven electronic forms as pre–assigned to him/her. After logging–in, s/he was asked to enter personal details (name, area(s) of work, employment status (working/retired), institution, state/union territory, alternative email ID). S/he was then taken through a self–orientation power–point tutorial. The list of AOCs was then displayed on his/her computer screen and the participant was instructed to select any two AOCs to contribute RIs in the four domains of research (description, discovery, delivery and development). The expert was not limited in the number of RIs s/he could submit under each domain. The electronic forms allowed for completion over multiple sessions. An offline version of the form was prepared and shared with participants who had difficulty in accessing the internet. A total of 3497 RIs were obtained across the MNCHN themes from 498 experts (42.3% participation).

**Refinement of the research ideas:** The RPS project management team at INCLEN along with the RSCs closely examined each RI and rephrased, split, and combined the RIs (as required) keeping the core idea intact and without discarding any RI. The original RI list was maintained as a separate file for ready reference at any time. The process was intuitive, consultative and iterative (completed through brainstorming by teams over several sittings). As far as possible, the RIs were refined in a way that described the population, intervention, control, and outcome (PICO). This process led to a compendium of 4003 RIs from the original 3497 RIs. (**Table 5**).

**Table 5:** Research ideas obtained through the first round of crowd–sourcing and subsequent refinement

Theme	Component	Total number of areas of concern	Number of research ideas (received)	Number of research ideas (after refinement)
Maternal health	Mortality	13	436	523
	Stillbirths	15	418	542
	Morbidity	8	353	243
	Lateral submissions*		11	**–**
	Subtotal		**1218**	**1308**
**Newborn health**	**–**	8	**641**	**626**
**Child health**	**–**	12	**596**	**648**
**Nutrition**	Maternal nutrition	12	450	590
	Childhood nutrition	16	590	831
	Lateral submissions*		2	**–**
	Subtotal		**1042**	**1421**
	**Total**		**3497**	**4003**

*Research ideas received from the National Steering Group as and when through hand–written submissions.

**Development of research options (ROs):** The 4003 RIs were consolidated onto 373 ROs. These were crystallized through iterative refinement to avoid duplication and redundancy. Each RO represented a portfolio of inter–related RIs that addressed a central research concept. Thus, the ROs addressed multiple AOCs and several of these pertained to cross–cutting issues across domains, components and themes. The ROs were finally categorized into four themes (maternal health: 122, newborn health: 56, child health: 101, nutrition: 94) (**Table 6**).

**Table 6:** Distribution of the research options in the domains of research

Domain of research	Frequency (%) of research options in themes			
**Maternal health**	**Newborn health**	**Child health**	**Nutrition**
Description	42 (34.4)	15 (26.8)	39 (38.6)	35 (37.2)
Delivery	57 (46.7)	24 (42.9)	37 (36.6)	42 (44.7)
Development	44 (36.1)	21 (37.5)	37 (36.6)	27 (28.7)
Discovery	8 (6.6)	4 (7.1)	4 (4.0)	2 (2.1)
Single domain	29 (23.8)	8 (14.3)	16 (15.8)	12 (12.8)
>1 domain	93 (76.2)	48 (85.7)	85 (84.2)	82 (87.2)
Total (N = 373)	122 (100.0)	56 (100.0)	101 (100.0)	94 (100.0)

##### Second round of crowdsourcing

**Finalization of criteria for scoring:** Previously published CHNRI exercises were reviewed extensively to retrieve scoring criteria used in past exercises. Two rounds of consultation were held with RSC members, international CHNRI experts, and experts from the World Health Organization who had been closely associated with previous CHNRI exercises. Five succinctly worded criteria (answerability, relevance, equity, innovation and out–of–the–box thinking, and investment on research) were finalized. These criteria were believed to be consistently applicable across domains, themes and ROs (**Box 2**). The context and scope of the present exercise, nature of the ROs and the large number of scorers from various disciplines across India that were to score the research options were the key considerations while deciding on the scoring criteria to be used. The scorers were expected to evaluate the ROs against the criteria by choosing one of the following responses: *‘Yes’* if the research option favorably met the criterion query, *‘No’* if it did not, and *‘Not my expertise’* if the scorer felt that s/he was not sufficiently informed to adjudge the research option against the particular criterion. While other CHNRI exercises employed sub–questions under each criterion, we chose to forego sub–questions as we were advised that sub–questions usually had high agreement [12] and also because our exercise had a large number of ROs to be scored and we were aiming to maximize retention of participants by minimizing scorer fatigue, especially in order to preserve the validity of our planned regional analyses.

Box 2 **Scoring criteria and their definitions**

1. **Answerability.** Can the research be done through ethical, transparent, well–designed, “do–able” studies with the existing local and national capacities and or by strengthening the existing capacities through regional or global collaboration?
2. **Relevance.** Is it likely that the research would address a high burden condition and critical gap in knowledge?
3. **Innovation and out–of–box thinking to resolve complex, and refractory challenges.** Does the new research have the potential for transformative change in the health system/ health care?
4. **Equity.** Is it likely that the research product will address the differences in health and nutrition that are systematically associated with social, cultural and economic hierarchies, ethnicity, gender, environment and geographic disadvantages, thereby reducing inequities?
5. **Investment on research.** Is it likely that the potential impact and benefits of the new knowledge on health/ nutrition will outweigh the consideration of investments on research?

**Scoring of the research options by the Nationwide Network:** The scoring exercise was done using a user–friendly online interface *(www.surveymonkey.com)* that allowed for having individualized scorer accounts that could be accessed through an invitation email from the INCLEN RPS project management team. The software could readily archive access details (email and IP addresses) and responses selected by the scorer. Once the scorer logged in, s/he underwent a comprehensive orientation of the context and method of the exercise, and the scoring criteria and process. Thereafter, ROs appeared in a random sequence, one at a time, on the scorer’s computer/smart phone screen. The scorer was requested to score all the ROs for the assigned theme. As the number of ROs to be scored was high and could have led to high scorer burden and attrition, each scorer was randomly allocated a combination of two of the five criteria for scoring. Five such criteria combinations (survey questionnaires) had been prepared for scoring: (i) Answerability and Innovation; (ii) Answerability and Equity; (iii) Relevance and Innovation; (iv) Relevance and Investment on Research; and (v) Equity and Investment on Research. The nationwide network was stratified at two levels: first, according to their participation status in the first round of crowd sourcing (‘participated’, ‘could not participate’, or ‘newly invited’ experts); and, second, according to their region. Subsequently, the experts within each region were equally distributed across the five survey questionnaires within the theme through consecutive allocation (the expert with serial number 1 got Survey Questionnaire 1; the next in line got Survey Questionnaire 2 and so on; the questionnaire allocation cycle was restarted with every 6th expert).

It was mandatory for the scorer to evaluate the RO on the screen against both of the assigned criteria before moving on to the next RO (ie, skip logic was disabled). However, the scorer could review and edit his previous responses once s/he had moved forward. Completion over multiple sessions was allowed to avoid effects of scorer fatigue and overcome time constraints. The RPS project management team at INCLEN remained vigorously engaged with the nationwide network through email and telephone for immediate troubleshooting and timely reminders, and used continuous real–time data monitoring to check progress. Scorers who requested hard copies of the questionnaires instead of the online process were provided with the same for recording the responses. In the second round of crowdsourcing, 893 scorers participated (58.1% participation rate) (**Table 7**).

**Table 7:** Distribution of experts who participated in the 2^nd^ round of crowd–sourcing (the Scoring Exercise)

Region	Maternal health			Newborn health			Child health			Nutrition			Overall		
**Male**	**Female**	**Total**	**Male**	**Female**	**Total**	**Male**	**Female**	**Total**	**Male**	**Female**	**Total**	**Male**	**Female**	**Total**
EAG States and North Eastern States	39	48	87	39	23	62	55	14	69	42	28	70	175	113	**288**
Northern States and UTs (including West Bengal)	25	44	69	57	15	72	52	16	68	31	29	60	165	104	**269**
Southern and Western States and UTs	39	55	94	37	27	64	69	27	96	32	50	82	177	159	**336**
**Total**	**103**	**147**	**250**	**133**	**65**	**198**	**176**	**57**	**233**	**105**	**107**	**212**	**517**	**376**	**893**

EAG – Empowered Action Group, UT – Union Territories

#### Assignment of relative criteria weights by the LRG

The LRG members were given an in–depth explanation of the CHNRI exercise. They were then requested to assign relative weights to the scoring criteria by distributing a hypothetical amount of Indian Rupees (INR) 100 across the five criteria, giving the maximum amount to the criteria they felt to be the most important and the minimum to the least important. The relative weight for each criterion was computed by calculating the arithmetic mean of the average amount received by the respective criterion in each LRG constituency (**Table 8**). Of 84 members approached for the LRG, 79 participated (94.0% participation). The LRG ascribed maximum relative weight to Relevance (0.254), followed by Innovation and Out–of–Box Thinking (0.199), Equity (0.193), Answerability (0.192), and Investment on Research (0.161).

**Table 8:** Relative weights assigned to the scoring criteria by the Larger Reference Group

LRG categories	Answerability	Relevance	Equity	Innovation	Investment on research
Policy decision makers, politicians (N = 18)	0.197	0.229	0.209	0.203	0.162
Eminent researchers (N = 17)	0.212	0.245	0.169	0.197	0.177
MNCHN program managers from central and state governments (N = 24)	0.186	0.254	0.201	0.198	0.162
Funding agencies (N = 20)	0.173	0.288	0.195	0.200	0.145
**Overall (N = 79)**	**0.192**	**0.254**	**0.193**	**0.199**	**0.161**

LRG – Larger Reference Group, MNCHN – Maternal, Newborn, Child Health and Nutrition

### Data management and analysis

The *“Yes”* and *“No”* responses were scored as “1” and “0” respectively. The *“Not my expertise”* responses were excluded from the calculations. Relative ranking and Research Priority Scores (RPS) were calculated as follows [13]:

Average scores received against each of the five criteria were calculated for each RO.

1. The criteria weights (as assigned by the LRG) were applied to the mean score received by each criterion.
2. Research Priority Scores (RPS) were calculated by adding together each criterion’s weighted scores for each RO.

The ROs were arranged in descending order of their RPS to get national and regional rankings. Work location of the scorer as entered by him/ her at the time of the scoring determined the regional ranking.

Average Expert Agreement (AEA) [14] was also calculated for each RO. The AEA is a proportion of scorers who scored the most common score for a particular RO divided by the total number of scorers who scored that RO.

The second meeting of the NSG reviewed the ranked list of national and regional research priorities. The group further suggested to identify ROs relevant to three more themes: (i) adolescence; (ii) issues cutting across four MNCHN themes for greater impact on health and health systems; and, (iii) areas requiring biotechnology methods from the compendium of 373 ROs, and generate ranked lists according to their RPS for each of these.

The results from all exercises are reported in–depth separately in manuscripts prepared for submission to the Journal of Global Health. The overall discussions by the National Steering Group on the results and way forward for the exercise have been has been accepted for publication in the *Indian Journal of Medical Research*.

## DISCUSSION

The COHRED Working Group on Priority Setting highlighted that engagement of a wide spectrum of stakeholders is essential to identify priorities that reflect research needs, available technical and financial capacity, and societal values and ethics [15]. Stakeholder engagement, and data and capacity constraints frequently impeded the process for setting priorities, more so in the LMICs [16]. The current exercise, through systematic inclusion of diverse range of national stakeholders in a LMIC setting, identified priorities for maternal, neonatal and child health and nutrition at national and sub–national (regional) levels. The exercise leveraged the inherent flexibility of the systematic CHNRI method and built further methodological robustness. CHNRI exercises hitherto had taken a conservative approach in considering active contribution to research/policy as a selection pre–requisite for scorers. In contrast, we expanded the stakeholder base to include diverse range of doers and users (techno–managerial) of research in the field of MNCHN. This helped in including a variety of viewpoints in the scoring process and possibly, led to prioritization of ROs that was important to both.

Having Indian nationals as the exclusive contributors and scorers to this exercise makes it unique from previous exercises. In this way, this CHNRI exercise is truly a representation of, and driven by, India’s health and nutrition community. Moreover, the exercise is the first to conduct su–national–level analysis which, in a country as large and diverse as India, is imperative to truly explore research priorities and enable the country to tailor interventions regionally. With effective use of technology and building on INCLEN’s network for multi–centric studies, 498 experts from across India contributed research ideas and 893 experts were involved in the scoring process. About 75 (60–96) experts were involved per region per theme to score the ROs. The large number of scorers (“sample size”) should have led to saturation and stable estimate of priority ranks at national and sub–national (regional) levels [8]. The improved response rates between first and second rounds of crowdsourcing should have reduced bias [17]. Gender distribution of scorers is a reflection of skewed gender participation in program management, research and academia for the themes considered in this exercise. The scorer profiles have been discussed in details in the respective thematic papers prepared for submission to JoGH.

To minimize scorer fatigue, we asked the participants to score against predefined pairs of criteria allocated randomly to them instead of all five criteria. The AEA for each evaluated research option represents the proportion of scorers that gave the most frequent (modal) response [14]. For the top 10 ROs at national level across the themes, the AEA for both individual and aggregate of the five criteria was fairly high (maternal health: 0.887—0.929; newborn health: 0.871—0.902; child health: 0.899—0.923; nutrition: 0.869—0.923) indicating consistency among the scorers. This also indicates minimal bias due to partial criteria scoring adopted in the current exercise and appears to be a pragmatic approach for better participant compliance without affecting the validity of the priority setting scoring. There were four distinct constituencies among the LRG; the LRG is to be viewed as a strength since different constituencies are likely to have differences in their collective perspective about research priorities [18]. It was interesting to observe that “Relevance” was accorded the highest weight by all the LRG sub–groups highlighting that priorities should be suited to the context.

In view of the disease burden and significance of the health systems in the implementation and delivery of services, the NSG suggested developing ranked priority lists for adolescent health, cross cutting themes and biotechnology related ROs from the 373 ROs spread across different themes. These lists will, at best, be an indicative priority list because the ROs were picked up from different thematic groups, scored by dissimilar set of experts with differences in their professional expertise. Although the overall AEA was high across themes, the validity of RO scores to determine their relative ranking shall remain unknown for these additional lists.

The exercise was the largest to–date use of the CHNRI methodology in terms of research ideas collected, processed and scored, and the number of participants and spectrum of stakeholder constituencies engaged. It expanded on the CHNRI methodology and thus, contributes to further evolution of the CHNRI method as a robust, inclusive, participatory, transparent and objective technique for identification of research priorities. It has been opined that prioritization processes will have an impact only if funders have a buy–in. It is also anticipated that there is an imminent challenge to develop tools to detect and evaluate the impact of CHNRI exercises on funder decision making and priorities [19]. A recent article in Lancet affixes with the research funders and research regulators, the primary responsibility of addressing the sources of avoidable waste once research priorities are set [20]. We hope that ICMR–INCLEN collaborative effort helps in rational distribution of health and nutrition research budget by the Government of India and donor agencies funding research in India and in similar LMIC contexts, and also inform any mid–course correction of currently funded research portfolio as needed. Sub–national (regional) prioritization should further help in matching the exercise’s findings to other LMIC contexts. This exercise can serve as a guidance for other LMICs, especially those with diversity among their populations, in setting research priorities nationally.
